# Modeling the dynamics of chromosomal alteration progression in cervical cancer: A computational model

**DOI:** 10.1371/journal.pone.0180882

**Published:** 2017-07-19

**Authors:** Augusto Cabrera-Becerril, Cruz Vargas-De-León, Sergio Hernández, Pedro Miramontes, Raúl Peralta

**Affiliations:** 1 Departamento de Matemáticas, Facultad de Ciencias, Universidad Nacional Autónoma de México, Ciudad de México, México; 2 Escuela Superior de Medicina, Instituto Politécnico Nacional, Ciudad de México, México; 3 Programa de Dinámica Nolineal, Universidad Autónoma de la Ciudad de México, Ciudad de México, México; 4 Centro de Investigación en Dinámica Celular, Instituto de Investigación en Ciencias Básicas y Aplicadas, Universidad Autónoma del Estado de Morelos, Cuernavaca, México; Universidade Estadual de Maringa, BRAZIL

## Abstract

Computational modeling has been applied to simulate the heterogeneity of cancer behavior. The development of Cervical Cancer (CC) is a process in which the cell acquires dynamic behavior from non-deleterious and deleterious mutations, exhibiting chromosomal alterations as a manifestation of this dynamic. To further determine the progression of chromosomal alterations in precursor lesions and CC, we introduce a computational model to study the dynamics of deleterious and non-deleterious mutations as an outcome of tumor progression. The analysis of chromosomal alterations mediated by our model reveals that multiple deleterious mutations are more frequent in precursor lesions than in CC. Cells with lethal deleterious mutations would be eliminated, which would mitigate cancer progression; on the other hand, cells with non-deleterious mutations would become dominant, which could predispose them to cancer progression. The study of somatic alterations through computer simulations of cancer progression provides a feasible pathway for insights into the transformation of cell mechanisms in humans. During cancer progression, tumors may acquire new phenotype traits, such as the ability to invade and metastasize or to become clinically important when they develop drug resistance. Non-deleterious chromosomal alterations contribute to this progression.

## Introduction

The genome and chromosomal unbalance of a transformed cell is highly heterogeneous, with a wide range of structural and copy number alterations. On the other hand, the behavior of the tumor as a whole results from the accumulation of altered cells [1]. In particular, in Squamous Cell Carcinomas (SCC), there is strong experimental evidence for a correlation between copy-number unbalance and tumor aggressiveness [2]. In this regard, a large number of chromosomal alterations could characterize an aggressive tumor, while a smaller number of these alterations could be associated with a less aggressive tumor [3]. Therefore, finding the progression of these alterations would allow us to address the cancer treatment based on the profile of chromosomal alterations.

On the other hand, although all cells can undergo chromosomal alterations, only a small number of such alterations have the potential to be deleterious to the cell, while the majority of chromosomal alterations are not deleterious [4]. In this context, the development of cancer is the result of the accumulation of several non-deleterious mutations. In the early stages of cancer, non-deleterious alterations are few; however, in advanced stages, these alterations are considerable [5]. Several studies in molecular cytogenetics (e.g., comparative genomic hybridization) have investigated chromosomal alterations in cancer. Some of the authors of these studies correlate these chromosomal alterations with specific tumor behaviors [6]–[9].

Cervical Cancer (CC) is the second most common malignancy in women worldwide. Infection with high-risk Human Papillomavirus (HR HPV) is the major etiological factor for this tumor [10]. HR HPV can transform infected cells through the direct action of the products of two of its early genes: E6 and E7. The E6 and E7 proteins of HR HPV are able to interact with molecules important to growth regulation and cell replication and can repair damage to the DNA of healthy cells. The E6 protein of HR HPV binds with high affinity to the molecule known as p53, inducing its degradation. The p53 protein is an important regulator of cell replication and is known as the main tumor repressor in humans; p53 is able to detect damage to DNA and arrest cell replication. A high proportion of human cancers have been shown to have damage in the gene encoding the p53 protein; CC is an exception to this because in this case, the gene is intact, but the protein is not present in the cells infected by HR HPV, and E6 has been responsible for removing it. In this case, the cell cannot repair DNA errors and will experience tumor development when the number of mutations increases. On the other hand, the E7 protein binds specifically to the tumor repressor gene product Rb. This gene was discovered and characterized in retinoblastoma [11]. This is a cell-cycle regulatory factor and is directly linked with the E2F transcription factor that, in turn, induces the transcription of elements involved in cell replication. The E7 protein of HR HPV possesses a high affinity for Rb-E2F. When the E7 protein binds to Rb, E2F is released and induces cell proliferation. Thus, E6 and E7 cooperate efficiently in cell transformation, stimulating chromosomal alterations in the uterine cervix. The profile of chromosomal alterations is very heterogeneous in precursor lesions (cervical intraepithelial neoplasms 1, 2, and 3), but this heterogeneity decreases in CC. In advanced tumors, we observe a profile of chromosomal alterations induced by HPV oncoproteins [11, 12]. Using computational tools, it is possible to obtain a model of the progression of chromosomal alterations from the appearance of precursor lesions until CC [13]–[18]. Within the context of a cancer, these models help in determining the global behavior of the tumor [19, 20]. The aim of this study is to determine the progression of chromosomal alterations, including deleterious or non-deleterious alterations, from precursor lesions to CC, using a computational model.

## Computational model of cervical cancer

The molecular biology methods applied to study chromosomal alterations in CC indicate a great heterogeneity of such alterations. Thus, CC behaves as a complex system, rendering computational tools ideal for the study of the behavior of this tumor.

Agent-Based Modeling (ABM) is a computational modeling technique for the study of complex systems, i.e., systems that are composed of many interacting elements. The main idea of ABM is to replicate, with a stimulus, some of the interactions among individual components of the system. ABM consists of the following three main components: agents, rules that govern interactions among agents, and the environment in which agents interact. Currently, there are many options and platforms for developing ABM; however, it has only recently been applied in the study of cancer [21–23]. For example, in a biological system such as tissue, each cell can be represented as an agent, and these agents may receive signals and input from the environment and their neighboring agents, providing output to the environment and their neighbors and making decisions based on input from their surrounding environment and from their internal signals. Within this context, the dynamics of chromosomal alterations, from precursor lesions to CC, can be modeled by ABM. Under conditions in which a deleterious mutation is present, cells enter into an apoptotic state. If a non-deleterious mutation is active, the cell progresses to the development of advanced lesions and cancer. There are at least three advantages of utilizing ABM as a mechanism for modeling cellular cancer: multi-scale modeling, randomness, and emergent behavior. Additionally, we can employ a random distribution to simulate external stimuli and to account for stochastic effects, which are always present in biological phenomena.

On the other hand, with Cellular Automata (CA), it is possible make idealizations of physical systems, as they are discrete dynamical systems both in time and in space. For example, a single cellular automaton consists of a line of cells, each with a value of 0 or 1 (true or false). These values are updated in a sequence of discrete time steps according to a definite, fixed rule [24]. CA produce complex behavior even with the simplest defining rules. In general, cells have a finite number *k* of possible values and may be arranged on a regular lattice in any number of dimensions. Some defining characteristics of CA are as follows: they are discrete in space and in time; they have discrete states; they are homogeneous; and they allow for synchronous updating. The rule of each cell depends solely on the values of a local neighborhood of cells around it, and its state depends only on its values in the preceding steps. Many biological systems have been modeled using CA [14, 15]. The development of structure and patterns in the growth of organisms often appears to be governed by very simple and local rules; therefore, they are well-described by CA. The advantages of using CA for modeling is that this method may be treated as parallel processing computers; thus, complex behavior that involves many individual cells can be properly modeled with CA. Cervical cancer has been modeled in different works using CA combined with another techniques [16]. In this work, we model cellular behavior using CA and cellular dynamics with ABM to determine the dynamics of deleterious and non-deleterious alterations in cervical tissue; therefore, our agents are autonomous, probabilistic-state cellular automata.

## Overview of the model

**Fig 1:**
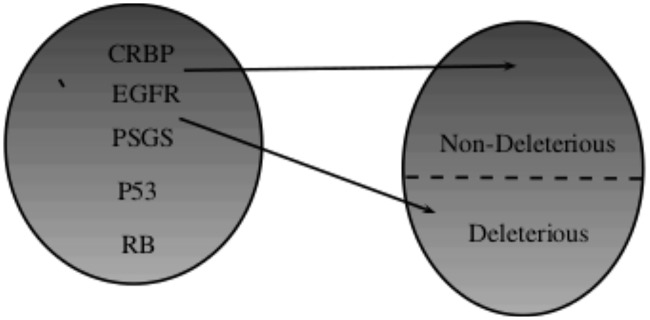
Minimal chromosome alterations in a cell.

The “ABM-Cervical-Cancer” model is a hybrid, two-Dimensional (2D) computational model implemented in the Netlogo framework [17]. This model consists of a set of agents that simulate cell behavior. We decided to construct each cell with a minimal chromosome that is subjected to two possible alterations, deleterious and non-deleterious mutations, and each gene has three possible states: silenced; normal, or overexpressed. Fig 1.

We introduce the global variable “HPV” as a Boolean variable. When HPV is true, there is infection with HPV, integration in the genome, and the triggering of random alterations, behaviors that are suggested by the literature [25].

The variable *P* simulates the clinical manifestation of HPV infection, and *P* is the outcome of the roll of a pair of dice: if the result is greater than or equal to 2 (*P* ≥ 2), the HPV-infected host will probably exhibit a Cervical Intraepithelial Neoplasia (CIN)1 lesion; if the result of the roll of the dice is greater than or equal to 5 (*P* ≥ 5), it is more probable that a CIN1 lesion will evolve into a CIN2 lesion; and if *P* ≠ 7, it is highly probable that the host will develop a CIN3 lesion and, once in this state, develop Cervical Cancer (CC) with probability *η*.

**Fig 2:**
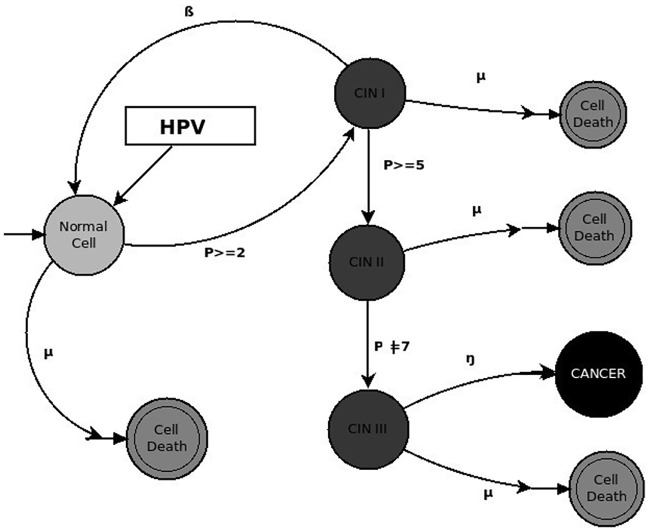
The ABM-Cervical-Cancer model.

The evolution from HPV infection to CC is simulated by counting the number of cells that present early-stage cervical intraepithelial neoplasia lesions. A second roll of the dice yields the probability for progressing from CIN1 to CIN2, although some of the cells with CIN1 lesions will likely recover, with probability *β* = 0.01. If some deleterious alterations are present, cell death will be more probable and cancer will not develop. The “natural” cell-death rate is *μ*. Individual cells have two forms of interaction: Each cell breeds a new cell when it reaches a “mature” stage (when it passes a fixed time *τ*), and the offspring will inherit the chromosome of the “parent-cell”. When a large number of cell neighbors are cancerous, the probability of progressing from CIN2 to CIN3 or to cancer is increased Fig 2. In the S1 Appendix, we have included a detailed description of the model using the ODD (Overview, Design concepts and Details) protocol and the full code for the implementation in NetLogo in S1 File.

**Fig 3:**
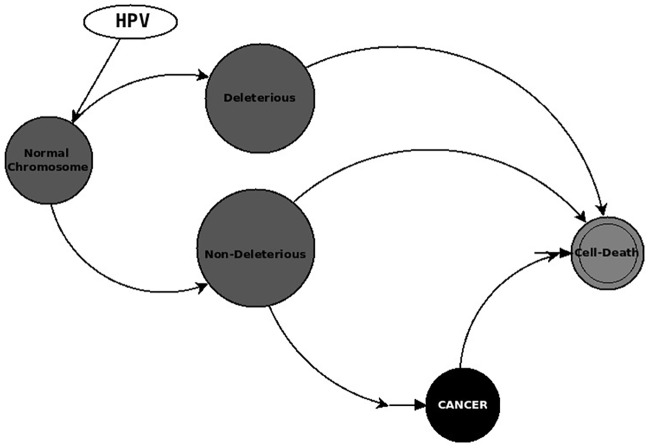
Chromosomal alteration dynamics.

**Fig 4:**
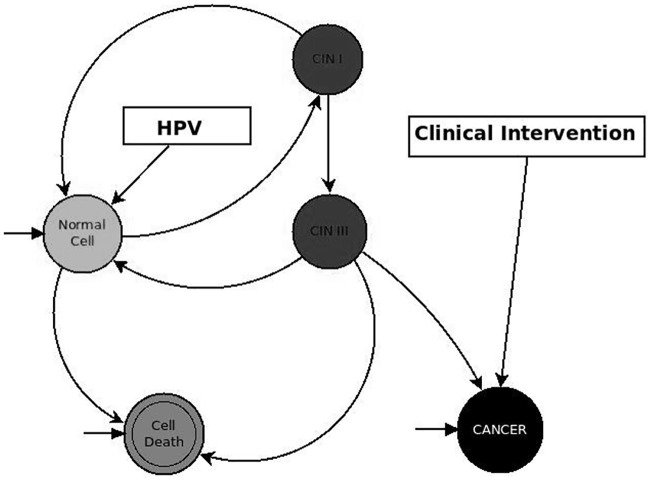
Dynamics of the ABM-Cervical-Cancer model with the Clinical Intervention variable.

In our model, the Clinical Intervention variable is an external stimulus that randomly selects a patch of cancer cells and deletes it in one step of the simulation; this allows us to simulate real clinical intervention. In the next step, the dynamics repeat; thus, cancer cells remain present after Clinical Intervention, but their growth is being bound. The mechanism produces loops in the simulation, as depicted in Figs 3 and 4. Additionally, the randomness produces oscillations in the dynamics of the model.

**Fig 5:**
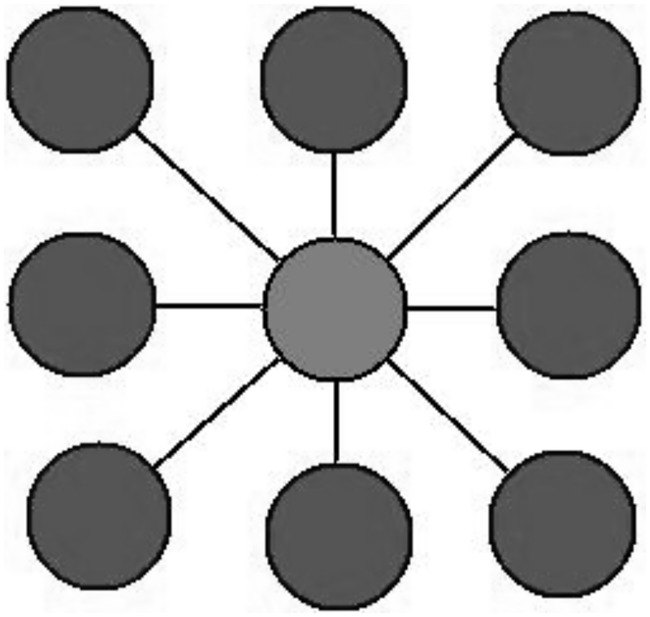
Neighborhood in the ABM-Cervical-Cancer model.

**Fig 6:**
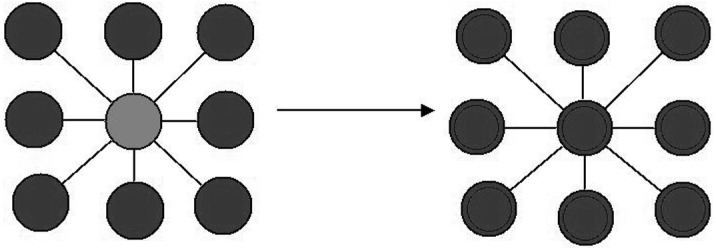
“Horizontal” interaction.

**Fig 7:**
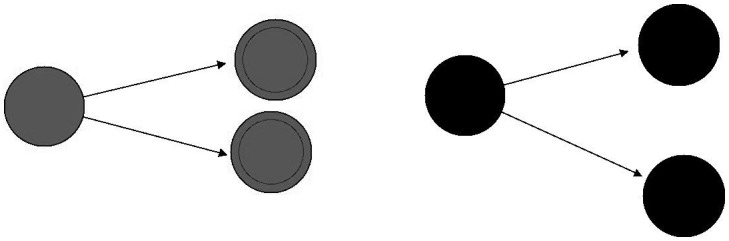
“Vertical” interaction or mitotic transmission.

The cells possess two means of interaction: “horizontal” interaction and “vertical” interaction. Vertical Interaction refers to the inheritance of characteristics or mitotic transmission. Horizontal Interaction occurs with the nearest cells in a De Moore neighborhood. Horizontal Interaction is governed by a random process; thus, the probability for a single cell to develop cancer increases when cancer cells are nearby in its neighborhood (Figs 5, 6 and 7).

The condition for halting the program consists either of the tissue comprising 75% cancer cells or reaching 20,000 steps in the simulation. It is worth noting that in this model, we do not pretend to simulate the growth of cancer masses; rather, we simulate chromosomal variations as the cancer cells grow. Therefore, the movement of the agents does not correspond to the natural dynamics of tissue growth, but rather, chromosomal alterations are simulated carefully.

## Simulation and results

We define two different experiments, each consisting of 100 runs with 20,000 iterations of the ABM-Cancer model in the NetLogo framework, with some initial conditions for the density of cell growth and cell death rate set at fixed values. For the initial experiments *E*_1_ and *E*_2_, the Clinical Intervention variable has been set as false. For experiments $E_{1_{1}}$ and $E_{2_{1}}$, this variable has been set as true. The main difference between the *E*_1_ and *E*_2_ experiments is the choice of values for cell density and death rate, as shown in the Table 1.

**Table 1:** ABM-Cervical-Cancer model initial setup for numerical experiments.

Experiment	HPV	Cell count	Cell death rate	Clinical Inter
*E*_1_	true	1740	0.01	false
*E*_2_	true	6000	0.02	false
$E_{1_{1}}$	true	1740	0.01	true
$E_{2_{1}}$	true	6000	0.02	true

Experiments *E*_1_ and *E*_2_ showed similar statistical behavior. In 18% of the runs of experiment *E*_1_, CIN1 lesions appear, which is consistent with the 16% prevalence of cervical cancer due to HPV infection, as reported in the literature [26, 27]. Experiment *E*_2_ reveals a 12% prevalence of CIN1 lesions. Both experiments showed a different mean duration, while nearly all of the CIN1-positive cases in *E*_1_ had invasive cancer. In experiment *E*_2_, almost none of the runs presented invasive cancer (Table 1).

On the other hand, runs in which Clinical Intervention was set as true exhibited a minor prevalence of CIN1 lesions, as expected. In experiment $E_{1_{1}}$, there was an 11% prevalence, but in experiment $E_{2_{1}}$, we obtained a 12% prevalence, the same as in *E*_2_ with the absence of Clinical Intervention. Experiment $E_{2_{1}}$ exhibits interesting behavior: although Clinical Intervention is present, in cases where CIN1 lesions appear, invasive cervical cancer is more likely to develop; therefore, the simulation does not reach 20,000 steps, which may be a pattern caused by reaching the charge capacity of the system.

In Table 2, we show the arithmetical means of the results of the numerical experiments in the ABM-Cervical-Cancer model, summarizing the statistical behavior of the model.

**Table 2:** Statistical results from the ABM-Cervical-Cancer model numerical experiments.

Experiment	Max Steps	CIN1 prevalence	Deleterious	Non-delet	Ratios
*E*_1_	6400	18%	297.18	317.12	1.03
*E*_2_	20000	12%	250.23	264.35	1.05
$E_{1_{1}}$	20000	11%	123.83	148.34	1.2
$E_{2_{1}}$	18	12%	86.5	102.5	1.1

**Fig 8:**
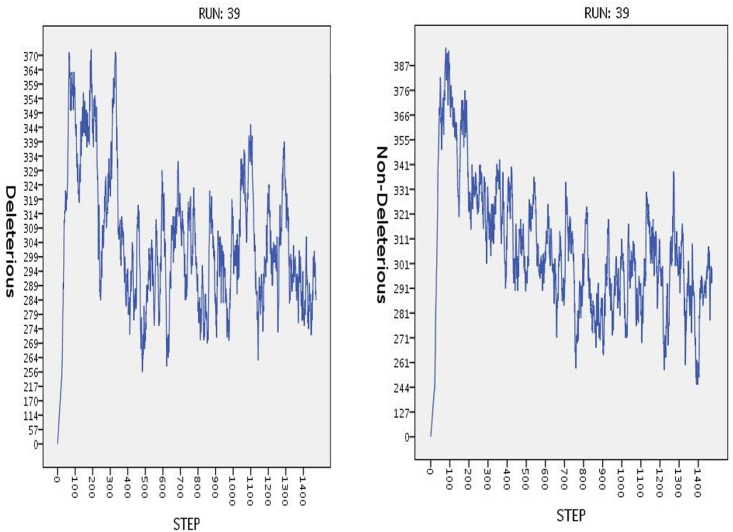
Time series for run 39 of experiment *E*_1_.

Dispersion analysis shows that there is a correlation between the heterogeneity of chromosomal alterations and cancer progression. An analysis of the time series shows that in cases where CC has developed, non-deleterious transformed cells are more prevalent than deleterious transformed cells. In subsequent figures, we depict some runs of experiment *E*_1_. Fig 8

In experiments *E*_2_ and $E_{1_{1}}$, simulations reached 20,000 steps, and in both cases, we can observe that an oscillation occurs in both deleterious and non-deleterious alterations. In experiment *E*_1_, we have a more interesting behavior: we have a noisy oscillation in both alterations, although in the case of $E_{1_{1}}$, the oscillations are bound due to Clinical Intervention. On the other hand, *E*_2_ exhibits an initial spike in both alterations, produced by the fast spread of the cancer cells, but eventually, the number of transformed cells reaches a stationary state. The main difference among these three experiments is that only in experiment *E*_1_ were we able to reproduce invasive cancer. Therefore, it is in this experiment that we expected to find the more interesting behavior.

**Fig 9:**
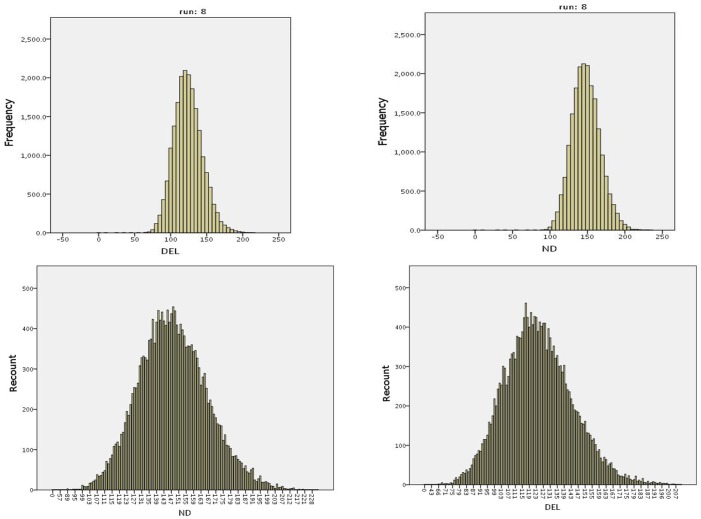
Histograms of frequencies and recounts in run 8 of experiment $E_{1_{1}}$. ND stands for non-deleterious, and DEL stands for deleterious.

In Fig 9, we present the histograms of the frequencies and recounts of deleterious and non-deleterious alterations for run 8 of $E_{1_{1}}$.

**Fig 10:**
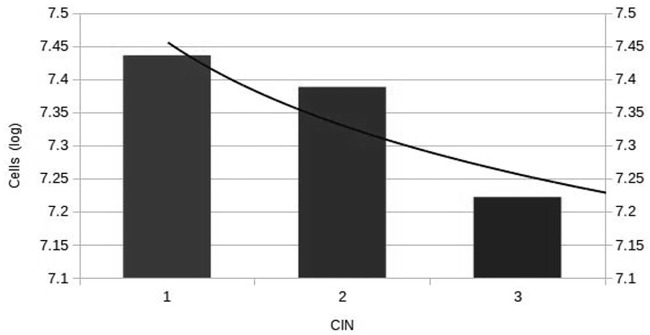
CIN lesion progression with deleterious alteration.

The progression of chromosomal alteration when non-deleterious mutations is present will probably drive the development of CC, acting as a selective mechanism; however, when deleterious mutations are present, cell death will probably rise. This can be observed in the results of our simulation. In the figure below, we illustrate the progression of CIN1 and CIN3 lesions and the progression of cervical cancer in run 39 of experiment *E*_1_ (we choose run 39 arbitrarily).Fig 10

## Discussion

The development of cancer can be seen as a process of accumulating mutations and chromosomal alterations, with the selection of cells that, to the best of their ability, adapt to their perturbed environment. The survival of the transformed cells is driven by the sequential accumulation of genetic alterations in sets of genes that control cell proliferation and the differentiation of signal transduction pathways. The number and types of somatic mutations accumulated during cancer progression are variable among different cancer types. Alterations typically include allele loss, point mutations, amplifications, and others, but these alterations are essentially divided between alterations that are deleterious and those that are non-deleterious to the cell [28].

In the oncology literature, the terms “driver genes” and “passenger genes” are widely used. Oncogenes and tumor suppressors are considered to be driver genes. Mutations in these genes result in the gain or loss of function and stimulate, either directly or indirectly, cellular survival and proliferation. Mutations that promote cancer development are considered driver mutations, and those that are not relevant in cellular transformation are called passenger mutations [29]. Most of the mutations present in the cell are passengers. However, as the rate of mutations increases, the cell acquires a mutator phenotype that promote cellular transformation. During this stage, the increase in the number of mutations in driver genes is evident [29]. On the other hand, in systems biology, specifically in the study of networks, the term “hub” is introduced as a central node. In this context, Palaniapan et al. defined hubs as highly connected nodes in a network, which can have a deleterious effect on the cell in the event that they are removed, whereas driver genes are those that promote tumor progression through the gain or loss of function (oncogenes and tumor suppressors, respectively) [30]. The human genome has drivers and passenger genes that can regulate the cell cycle, programmed cell death or apoptosis and other cellular processes that determine cellular fate. These genes fulfill the conditions of driver genes; mutations such as the loss or gain of function in driver genes are not deleterious to the cell. In this context, we can consider these mutations as non-deleterious in the same way as mutations are in passenger genes. Alterations in these genes change the genetic and epigenetic networks in cancer cells and reflect changes in the cellular processes defined above. In this framework, networks can be identified as the hubs or regions whose removal could be deleterious to the cell [30–32].

Deleterious regions may be important hubs that make multiple (functional or physical) connections with nodes that control tumor-cell behavior. It is currently not clear how the products of deleterious regions are organized into networks. However, recent evidence suggests that they may be organized as critical hubs in clusters among networks, with some of these mutated in the majority of cancers [25, 31, 33].

Some mutations are potentially deleterious to the cell, and in cancer progression, these do not accumulate. A cell with mutations could have both desirable (non-deleterious) and undesirable (deleterious) consequences. Cells with lethal deleterious mutations would be eliminated, which would mitigate cancer progression. However, cells with non-deleterious mutations would become dominant, which could predispose them to cancer progression. During cancer progression, it becomes increasingly harder to eliminate deleterious mutations and to fix beneficial mutations [34].

The types and number of documented somatic mutations in cancer are variable. The mutations that are selected promote tumor progression. These non-deleterious mutations help to explain why passenger and driver mutations are common in carcinomas. Interestingly, the frequencies of non-deleterious mutations accumulate in the transformed cells [35]. However, in our results, we found that deleterious mutations are more frequent in the early stages of cell transformation. Multiple deleterious mutations are most frequently found in the precursor lesion, which is invasive cancer [36]. Given the impossibility of human experimental manipulations, the analysis of somatic alterations using computer simulations during cancer progression provides a feasible pathway for generating insights into the transformation of cell mechanisms in humans. Somatic alterations can reveal much about the cancer cell during progression. During cancer progression, tumors may acquire new phenotype traits, such as the ability to invade and metastasize, or may become clinically important when they develop drug resistance. Acquired chromosomal alterations contribute to this progression [37].

The etiologic factor in cervical cancer and associated lesions is HPV infection. Two of the oncogenes of HPV, E6 and E7, induce cell transformation. At the clinical level, lesions associated with HPV infection are heterogeneous, and only one group progresses to invasive cancer. In this context, the HPV type that infects the cell is important in the malignant progression of the epithelium. HPV 16 infection is more likely to progress to cancer than infection with another genotype [16, 38]. The introduction of different specific HPV genotypes in our model could potentially modulate the progression of precursor lesions to CC.

Computational models such as Cellular Automata (CA), Agent Based Modeling (ABM), and their hybrids are in silico techniques for studying a variety of cancer behavior. Through computational programming, it is possible to simulate cellular behavior according to the type of mutations they present. Therefore, both CA and ABM have become powerful methods of modeling that are widely used by cancer researchers. There are many altered cellular processes in cancer, as well as the effects of different treatments, that have been modeled in computational systems [19, 39, 40]. Other mathematical-computational methods important in cancer research are network models. Network science has provided theoretical tools for understanding how the interaction of cellular components gives rise to cancer as an outcome of such interaction, such as in [32]. However, our approach does not consider all possible protein interactions but instead explores a plausible mechanism that relates chromosomal alterations to cancer development via emergent behavior of the cancerous cells.

In a hybrid model, each cell is often represented as an agent that behaves locally as a CA. Agents can receive signals from the environment and neighboring agents and make decisions based on these signals. In the context of cancer, an agent that simulates a transformed cell can grow or undergo apoptosis in response to surrounding environmental signals [19, 41]. During cancer progression, cellular proliferation requires genomic stability; if not, the cell will undergo apoptosis. In this regard, the dynamics of deleterious and non-deleterious mutations can be simulated as a manifestation of cell death mediated by apoptosis; i.e., deleterious mutations in precursor lesions are lethal to the cells. Thus, the latter do not progress to advanced lesions and cancer, while non-deleterious mutations in precursor lesions induce cellular transformations and cancer, rendering hybrid modeling an ideal tool to model this process.

## Conclusions

In this paper, we focused our efforts on the progression of chromosomal alterations in cervical cancer by employing a hybrid computational model and especially focused on the manner in which deleterious and non-deleterious alterations affect cancer behavior across different precursor lesions in CC.

Interestingly, this study revealed a significantly high frequency of deleterious mutations in precursor lesions and a low frequency in the mutations in CC. Precursor lesions of the cervix are well defined, and their progression to CC shows that it can be seen as an accumulation of deleterious mutations. Deleterious mutations are more common in precursor lesions, but these do not occur in later or advanced stages of cancer.

Some extensions of the model may include new variables, for instance, a vaccination variable. Vaccination could be simulated as another external stimulus that modulates the clinical manifestation of HPV infection; therefore, it would be a weight function of the P variable. We can also extend the model to simulate interactions among scales; thus, we can begin to elucidate how chromosomal alteration is connected to other phenomenological manifestations of cervical cancer by constructing a multi-scale version of the ABM-Cervical-Cancer model.

## Supporting information

S1 File Code for the NetLogo implementation of the ABM-CC model. (PDF)

S1 Appendix ODD for ABM-Cervical-Cancer ODD protocol for the ABM-CC model. (PDF)
