# Supplementary material for: Modeling the dynamics of chromosomal alteration progression in cervical cancer: A computational model
Source: PLoS One. 2017 Jul 19;12(7):e0180882. doi: 10.1371/journal.pone.0180882 (PMC5516994; doi:10.1371/journal.pone.0180882)
Supplement: S1 File — (PDF) [file pone.0180882.s001.pdf]

# NetLogo Code for Abm-Cancer.

```
globals[ dado dado2 n-cell n-cell-cn1 n-cell-cn2 n-cell-cn3 ncc n-cell-l  n-cell-del n-cell-ndel T P
turtles-own [crbp egfr psgs p53 rb cn1 cn2 cn3 cr clr edad]
patches-own [espacio]
;extensions [matrix rserve]
;extensions [r]


to setup
  __clear-all-and-reset-ticks
  set-default-shape turtles "circle"
  crt cuantos
  ask turtles [set xcor random 100  set ycor -100  set color red fd 1]
  ask patches [if (any?  turtles-here = true)[set espacio false]]
  ask patches [if (espacio = false)[ask one-of turtles-here [fd 1]]]
  set-patch-size 20
  resize-world -10 10 -10 10
  ask patches [set pcolor grey ]
  ask turtles [set crbp  0  set egfr  0 set psgs 0 set p53  0  set rb 0  set clr color set edad 0 se
  set n-cell count turtles
  set ncc 0
  set P (random 6 + random 6)
  set T 0
  set n-cell-del count turtles with [egfr > 0]
  set n-cell-ndel count turtles with [crbp > 0]
  show (random 2) - (random 3)
end

to go
  ask turtles [if (any? other turtles-here )[fd 1]]
  ask turtles [set edad edad + 1 ]
  ask n-of 80 turtles [divide]
  ask n-of 10 turtles [lesion_cn1 lesion_cn2 lesion_cn3]
  set n-cell count turtles
  set n-cell-cn1 count turtles with [cn1 = true]
  set n-cell-cn2 count turtles with [cn2 = true]
  set n-cell-cn3 count turtles with [cn3 = true]
  if  n-cell > 350 [muerte]
  CNR
  QT
  QIR
  grafica
  set T T + 1
  set n-cell-del count turtles with [egfr > 0]
  set n-cell-ndel count turtles with [crbp > 0]
```

```
  tick
  if T = 20000 or ncc >= floor (0.75 * n-cell)[ print ("fin de la simulación") stop]
end

to divide
  if edad >= 1 [ifelse vph_ = true [ifelse color != black  [hatch 1  [set crbp  ((random 2) - (rando
  set rb ((random 2) - (random 3))  set edad 0 fd 1 if (any? other turtles-here )[fd 1] set color re
  [ifelse tratamiento1 = true [if edad >= 6[die]][hatch 1[set crbp ((random 2) - (random 3))  set eg
  set rb ((random 2) - (random 3))  set edad 0 fd 1 if (any? other turtles-here )[fd 1] set color bl
  set clr color set edad 0 set cn1 false set cn2 false set cn3  false if (any? other turtles-here )[
end

to colorear
  ifelse crbp = 0 [set color clr][ifelse crbp > 0 [set color clr + random-float 3][set color clr - r
  ifelse egfr = 0 [set color clr][ifelse egfr > 0 [set color clr - random-float 3][set color clr + r
  ifelse psgs = 0 [set color clr][ifelse psgs > 0 [set color clr + random-float 3][set color clr - r
  ifelse p53 = 0 [set color clr][ifelse p53 < 0 [set color clr + random-float 3][set color clr - ran
  ifelse rb = 0 [set color clr][ifelse rb < 0 [set color clr + random-float 3][set color clr - rando
  if clr = white [set color 13]
end

to muerte
  ask n-of por_morir turtles [if edad >= 12  or (color = black and edad >= 8) or (p53 < 0 and edad >
end

to lesion_cn1
set dado  random 6
set dado2 random 6
if vph_ = true and (dado + dado2) = 2 [ask n-of (floor(0.3 * (n-cell)) )turtles [if (P != 7 and P >
end

to lesion_cn2
set dado random 6
set dado2 random 6
if vph_ = true and (dado2 + dado) = 5 [ask n-of (floor(3 * (n-cell-cn1))/(10)) turtles with [cn1 = t
end

to lesion_cn3
set dado random 6
set dado2 random 6
if vph_ = true and (dado2 + dado) = 7 [ask n-of (floor((3 * (n-cell-cn2))/(10))) turtles with [cn1 =
end

to grafica
  plot n-cell
  plot n-cell-cn1
```

```
  plot n-cell-cn2
  plot n-cell-cn3
end

to CNR
 set dado random 6
 set dado2 random 6
 ask turtles [if cn3 = true  and egfr > 0 and (dado + dado2) != 7 [set color black set clr color]]
 set ncc count turtles with [color = black]
end

to QT
  if tratamiento1 = true  [ask turtles with [cn3 = true][ifelse crbp >= 0 and egfr < 0 [set color re
end

to QIR
  if tratamiento2 = true [ask turtles with[color = black][die]]
end
```
