# Supplementary material for: Modeling the dynamics of chromosomal alteration progression in cervical cancer: A computational model
Source: PLoS One. 2017 Jul 19;12(7):e0180882. doi: 10.1371/journal.pone.0180882 (PMC5516994; doi:10.1371/journal.pone.0180882)
Supplement: S1 Appendix — (PDF) [file pone.0180882.s002.pdf]

Appendix: ODD for ABM-Cervical-Cancer

- Overview In some cases, the apparition of cervical cancer (CC) is preceded by three types of sequenced lessions (CIN1, CIN2 and CIN3) after the infection of HPV virus. The infection of HPV can produce chromosomal alterations. Progression of chromosomal alterations in cervical cancer has the main purpose of showing how different alterations, simulated by different values of random variables as external stimuli over the selected chromosome and the action of medical stimuli as well, can induce cervical cancer

- State Variables and Scales

  – Chromosome: Is an ordered pair (x,y) that will simulate the affected chromosome, it that reflects each of the stages in the model, x will reflect the appearance of cervical cancer (CC) and y will reflect the appearance of cervical cancer. The values that x can take models the different lessions that are associated previous to the appearance of CC and determined by a random variable, and another random variable will govern the state of y, that is either cancer is present or not. HPV is a Boolean variable that when set to True means that an infection of HPV on the tissue that can trigger random alterations, False otherwise

  – Clinical Intervention is a Boolean variable that when set to True, altered tissue cells are removed physically

  – P is a variable that simulates the strength of the host immune system. It is a result of a double dice rolling where the random variable (X) is the sum of the "visible" faces of the dice. If $X \geq 2$ then the host is infected with HPV and and then in a Bernoulli process we will determine if it does gets infected. CIN1 lessions will appear if $X \geq 5$ then it is more probable that CIN1 will become a CIN2 and also if $X \neq 7$ then it is highly probable to evolve to a CIN3 lession and from this state develop Cervical Cancer.

- Process Overview and Scheduling

  The evolution of HPV to CC is simulated counting the number of cells that present early stage and then another dice rolling will determine the probability of transition from CIN1 to CIN2. Figure 2 shows the schedule and transition of the model.

  In figure 3 it is shown the gene alteration dynamics that occurs when the cell is infected with HPV. The figure shows the transit to apoptosis when deleterious mutations occurs, but when non-deleterious mutations occurs we can have the proliferation of cancerous cells.

  In figure 4 it is shown the model when the Clinical Intervention variable is set to true and how the removal of cancerous cells will occur at different stages of the model.

- Design Concepts

  Since this model is thought to examine the relationship between the presence of CC given a different set of lessions and how this CC evolves in the presence of Clinical Intervention, the design concepts required to be mentioned to this regard are as follows.

  A double random process is required in order to pass from a specific type of lession to another, when the chromosome can present some type of lession then another random variable will determine if this lession (or CC) will be presented or not.

Some of the cells with CIN1 lession will probably recover with probability of 1%. If deleterious alteration are overexpressed, death cell will be more probable and cancer will not develop.

Clinical Intervention is an external stimuli that randomly chooses a number of cancer cells an deletes them, and the process is repeated in a next step

- Details
  - Initialization

    The initialization is set with HPV false for all chromosomes and it will run over 100 runs over 20,000 iterations with the Clinical Intervention variable set to false, another experiment is made with Clinical Intervention variable set to True.
  - Input

    The model does not require specific input other than initialization values
  - Submodels

    There are no particular submodels used in ABM-CC
